# Grape Pomace Polyphenol Extract Alleviates Obesity in Mice and Improves Gut Microbiota and Short Chain Fatty Acids

**DOI:** 10.3390/foods14162823

**Published:** 2025-08-14

**Authors:** Yulei Han, Chenlu Yang, Xuelin Tian, Xueqing Shi, Hua Wang, Hua Li

**Affiliations:** 1 College of Enology, Northwest A&F University, Yangling 712100, China; 2 College of Enology and Horticulture, Ningxia University, Yinchuan 750021, China; 3 China Wine Industry Technology Institute, Yinchuan 750021, China; 4 Shaanxi Engineering Research Center for Viti-Viniculture, Yangling 712100, China

**Keywords:** grape pomace, obesity, gut microbiota, short chain fatty acid

## Abstract

With the development of people’s diets and working patterns, obesity is an increasingly serious health threat faced globally. Grape pomace is an important by-product generated during the wine production process which is rich in polyphenols. Polyphenols show promising potential in anti-inflammatory, antioxidant, and metabolic regulatory applications. Nevertheless, the effects of grape pomace polyphenols on obesity alleviation and their underlying mechanisms require further investigation. The results of this study indicate that grape pomace polyphenols exhibit a preventive effect against obesity caused by a high-fat diet (HFD), ameliorated gut microbiota dysbiosis, and improved gut short chain fatty acid (SCFA) levels. The present study employed comprehensive bioinformatics approaches to characterize gut microbial profiles in each experimental group, including: alpha and beta diversity analyses, phylum- and genus-level relative abundance analyses, Linear Discriminant Analysis Effect Size (LEfSe), and Pearson correlation analysis between gut microbiota and short chain fatty acids. Both grape seeds extract (GSE) and grape peel extract (GPE) reduced the elevated F/B ratio caused by HFD, raised the abundance of probiotics such as *Lachnospiraceae_NK4A136_group*, *Bifidobacterium*, and *Blautia*, and mitigated the increase of pathogenic bacteria Fusobacteria and Eschericha-Shigella caused by HFD. Moreover, *Lactobacillus*, *Faecalibaculum*, *Clostridium-sensu-stricto-1*, *Bifidobacterium*, *Blautia*, *Alistipes*, and *Dubosiella* may be regulated by GSE and GPE to produce SCFAs, alleviating obesity and metabolic disorders. In conclusion, our results suggest that GSE and GPE show remarkable efficacy in ameliorating obesity and modulating gut microbiota in mice, providing evidence to support the utilization of grape pomace as a metabolic regulator.

## 1. Introduction

The prevalence of obesity constitutes a significant worldwide health challenge. Global figures from the World Health Organization reveal that adult obesity prevalence has increased by over two-fold since 1990 [1]. This trend is closely related to the modernization process of the food industry. The vigorous development of the ultra-processed foods industry has led to a significant transformation in the modern diet structure. Many delicious and tempting foods are inseparable from high-fat and high-sugar ingredients and cooking methods. Notably, an imbalance between energy intake and metabolic requirements leads to excessive caloric accumulation, thereby resulting in obesity [2]. Obesity is often accompanied by diseases such as hyperlipidemia, nonalcoholic fatty liver disease, cardiovascular disease, and diabetes mellitus [3]. It also serves as a major inducement for such metabolic diseases, increasing the health risks for individual.

Grape pomace is a by-product of the winemaking process after filtration and pressing, which contains polysaccharides, pectin, insoluble proanthocyanidins, and phenolic compounds [4]. Representative polyphenol components in grapes and their products include flavan-3-ol, flavonol, anthocyanidin, and phenolic acid compounds, among others [5]. The biological activity exhibited by phenolic compounds remains a key focus of scientific investigation. The existing evidence shows that grape and wine polyphenol extracts have the effects of regulating glucose metabolism [6], regulating lipid metabolism [7], improving hypertension, and protecting cardiovascular health [8], but the underlying mechanism remains to be elucidated. Previous studies have extensively investigated the health-modulating effects of grape seeds and their representative monomeric constituents [9,10,11], but grape peel is rich in anthocyanins and other phenolic compounds [12,13], and the synergy between the composite components may have a better effect on health regulation, so in the present study, grape peel and seed polyphenol extracts were put to use treating high-fat diet mice. Wine grape pomace is a good source of grape skin and seed polyphenol complexes that can be easily obtained in large quantities. Studying the regulation of grape pomace polyphenols from this source on obesity can not only provide ways and evidence to prevent obesity, but also provide a better treatment channel for wine by-products and create economic value.

Research increasingly identifies the gut microbiota as a critical component in metabolic regulation. Polyphenols exhibit low bioavailability in the human body [14]. The majority undergo metabolism by gut microbiota in the colon, producing low-molecular-weight metabolites that are subsequently absorbed and utilized by the body [15]. Consequently, the influence of polyphenols on gut microbiota is a major research focus. Grape polyphenols have been reported to regulate microbial population structure [16]. Isabel grape by-products increase the number of probiotics in the human gut microbiota [17]. However, the mechanisms by which grape pomace polyphenols affect gut microbiota and their metabolites in obese individuals remain to be further elucidated.

In this study, C57BL/6J mice were employed to establish an obesity model and grape seed polyphenol extract (GSE) and grape peel polyphenol extract (GPE) were administered. We investigated the effects of grape pomace polyphenol extract on indicators of obesity, intestinal microbes, and fecal SCFA in mice. This research aims to provide evidence to support the role of polyphenols in improving obesity by regulating the gut microbiota.

## 2. Materials and Methods

### 2.1. Polyphenol Extract from Grape Pomace

The polyphenol extract of grape pomace used in this study was prepared and prepared by the team in previous research [18]. The pomace of *Vitis vinifera* L. (Dunkelfelder) was collected at the Caoxingzhuang Vineyard (Xianyang, China) after the pressing stage of winemaking. Following manual separation of grape seeds and peels, polyphenolic compounds were individually extracted using 70% (*v*/*v*) ethanol. The resulting solutions are grape seed extract (GSE) and grape peels extract (GPE), respectively. The obtained extracts were subsequently lyophilized (Freeze dryer, FD-1C-50, Kun Shan Ultrasonic Instruments Co., Ltd., Kunshan, China) and stored at −20 °C for future experimental use.

### 2.2. Animal Experiments and Design

The 24 (8-week-old male) C57BL/6J mice used in this experiment were all purchased from Xi’an Jiaotong University (Xi’an, China), and all mice were fed a standard diet for 7 d to adapt to the environment. The mice were maintained under controlled environmental conditions, with relative humidity of 40–60%, a temperature of 22 ± 2 °C, and a 12 h light/dark cycle. During the experiment, the mice could eat and drink freely.

After the adaptation period, four test groups were formed with six mice per group and three mice per cage by random allocation. Mice in the Control group were fed a standard diet (AIN 93M) (Table A1) and mice in the high-fat diet (HFD) group were fed a high-fat diet (TP23100) (Table A2). Mice in GSE and GPE groups were fed a high-fat diet supplemented with 400 mg/kg body weight (BW) of fermented GSE or GPE, respectively, (Table A5). The standard feed and high-fat feed used in the experiment were purchased from TROPHIC Animal Feed High-Tech Co., Ltd. (Nantong, China). The standard feed provided 3.6 kcal/g with 10% of total energy derived from fat (Table A3), while the experimental high-fat diet had an energy content of 4.5 kcal/g (Table A4). Mice in the GSE and GPE groups received oral gavage of GSE and GPE distilled water suspensions every other day. Concurrently, to eliminate errors introduced by gavage, both Control and HFD mice received identical volumes of distilled water via the same oral gavage protocol.

**Figure 1:**
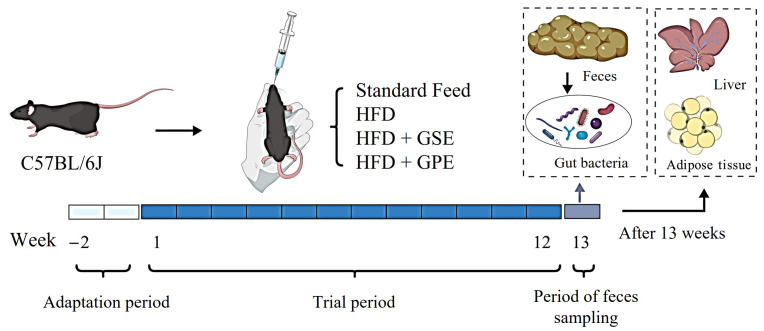
Schematic diagram of mouse experiment process. This schematic diagram was created with BioGDP.com.

After the adaptation period, the mice were fed according to groups, and their body weights were detected for 12 consecutive weeks. Fecal samples were taken during the 13th week (Figure 1) (Created with biogdp.com). After the 13th week, the mice were anesthetized after 12 h of fasting; blood was taken from the orbit. Blood samples were centrifuged (4 °C, 3000 rpm, 15 min) (Centrifuge, 5418R, Eppendorf AG, Hamburg, Germany) to isolate serum, which was transferred to −80 °C freezers. After the mouse was euthanized by cervical dislocation, we collected the colon, epididymal white adipose tissue, and liver, and promptly weighed the liver and epididymal adipose tissue. Some of the samples were put into 4% paraformaldehyde solution for fixation and used for histomorphological analysis, while the remaining tissue samples were encapsulated by the aluminum foil sterilized by high-temperature steam. Then, we immersed them in liquid nitrogen for quick freezing and transferred them to a −80 °C refrigerator for storage. The research adhered strictly to the standards set forth in the Guide for the Care and Use of Laboratory Animals (8th ed., ISBN-10: 0-309-15396-4). All experimental protocols received prior approval from the Experimental Animal Ethics Committee of Northwest A&F University (Permission ID: 20200528-010, Date: 15 November 2020).

### 2.3. Histomorphology Analysis

For histomorphology analysis, liver and epididymal white adipose tissue samples were fixed in 4% paraformaldehyde, embedded in paraffin, and sectioned (Ultra-Thin Semiautomatic Microtome (RM2235), Leica Biosystems, Wetzlar, Germany). Tissue sections were then stained with hematoxylin and eosin (H&E) and examined under a light microscope (Olympus, Tokyo, Japan).

### 2.4. Detection of Liver Biochemical Indices

Hepatic total cholesterol (TC), triglycerides (TG), serum aspartate aminotransferase (AST), and alanine aminotransferase (ALT) levels determined according to the manufacturer’s protocols of enzymatic assay kits (Nanjing Jiancheng Bioengineering Institute, Nanjing, China).

### 2.5. Collection of Fecal Samples

In the 13th week, fecal samples were collected by restraining the mouse after 12 h of gavage, lifting its tail, and applying gentle pressure to the lower abdomen. Fresh feces were directly collected into sterile EP tubes labeled with corresponding identifiers, then immediately sealed and stored at −80℃.

### 2.6. 16S rDNA High-Throughput Sequencing

Mouse fecal DNA was isolated using commercial kits (HiPure Stool DNA Kits, Magen, Guangzhou, China), adhering strictly to the provided guidelines. The nucleic acid samples were subjected to agarose gel electrophoresis to assess DNA integrity. Qualified nucleic acid samples were amplified for the 16S rDNA V3-V4 regions of using barcoded specific primers. Purification of amplified products was conducted with AMPure XP Beads, quantified with the ABI StepOnePlusReal-Time PCR System (Life Technologies, South San Francisco, CA, USA). Following purification, amplicons were pooled equimolarly and subjected to paired-end sequencing (2 × 250 bp) on an Illumina platform using standard procedures.

### 2.7. Bioinformatics Analysis

Following raw read acquisition from sequencing, low-quality reads were initially filtered prior to assembly. The paired-end reads were spliced into tags, the tag was then filtered and the resulting data was called a clean tag. Filtered sequences were clustered, and chimeras identified during cluster alignment were excised. The resulting high-quality sequences constituted the effective tags. After obtaining OTU, OTU abundance statistics were performed based on the effective tag.

Chao1, ACE, Shannon, and Simpson indices were calculated and visualized. Beta diversity was demonstrated by principal coordinates analysis (PCoA) and nonmetric multi-dimensional scaling (NMDS). Microbial biomarkers were identified using Linear Discriminant Analysis Effect Size (LEfSe). The association between SCFA and species is represented by the Pearson correlation coefficient. Correlational heatmaps and networks were constructed via Omicsmart, which is an interactive cloud-based platform enabling real-time bioinformatics analysis. (http://www.omicsmart.com, accessed on 1 January 2025).

### 2.8. SCFA Detection

To approximately 0.3 g of fecal sample, we added 1 mL of double distilled water. We ground the mixture at 4 °C (60 Hz, 15 s) (Ningbo Scientz Biotechnology Co., Ltd., Scientz-4807060206, Ningbo, China), then centrifuged it at 13,500 rpm and 4 °C until complete solid–liquid separation was achieved. We mixed the resulting liquid with 400 µL of metaphosphoric acid (AR) and left it to stand at 4 °C for 3–4 h. We centrifuged it for 15 min (13,500 rpm, 4 °C) to separate the precipitated proteins. We filtered the supernatant through 0.22 µm aqueous-phase filter membranes; subsequently, the filtrate was injected into the gas chromatography vials for analysis.

### 2.9. Statistical Analysis

Statistical analyses were performed using SPSS (Version 19.0). Intergroup differences were assessed by one-way analysis of variance (ANOVA), followed by Tukey’s honestly significant difference (HSD) post hoc test for multiple comparisons. A *p*-value < 0.05 was considered statistically significant. Mapping was performed using GraphPad Prism (Version 9.3.1).

## 3. Results

### 3.1. Body Weight and Organ Coefficient

**Figure 2:**
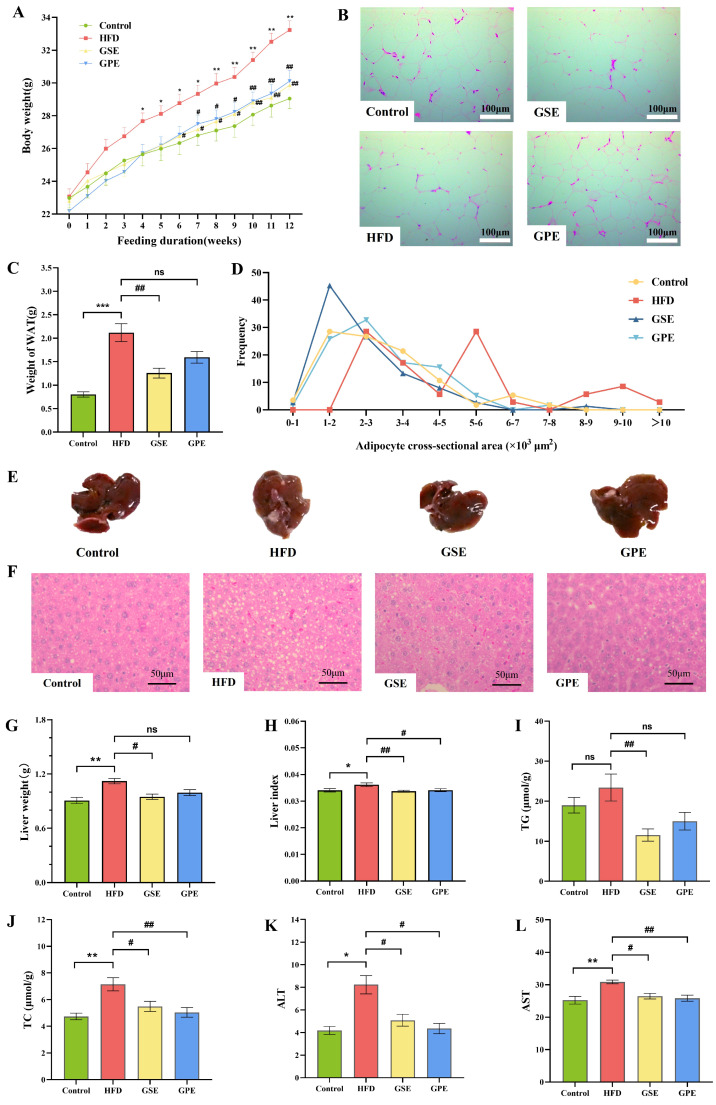
The effects of GSE and GPE on obesity-related phenotypes in HFD mice. (**A**) Body weight. (**B**) H&E-stained sections of white epididymal adipose tissue. (**C**) Weight of white epididymal adipose tissue. (**D**) Frequency distribution of adipocyte size in epididymal white adipose tissue. (**E**) Gross anatomical morphology of the mice liver. (**F**) H&E-stained liver sections. (**G**) Weight of liver. (**H**) Liver index. (**I**) Liver triglyceride levels. (**J**) Liver total cholesterol levels. (**K**) Serum ALT levels. (**L**) Serum AST levels. Data are presented as means ± SEMs (*n* = 6), * *p* < 0.05, ** *p* < 0.01, *** *p* < 0.001 versus Control group, ^#^ *p* < 0.05, ^#^^#^ *p* < 0.01 versus HFD group.

A significant increase in body weight became apparent in the HFD group relative to the Control group by the fourth week of dietary intervention. After the 6th and 7th weeks, the body weight of mice in the GSE group and the GPE group was significantly lower than that in the HFD group, respectively, (Figure 2A). High-fat diet (HFD) feeding resulted in significantly increased body weight in mice compared to Control groups, and grape pomace can control the high-fat-diet-induced excessive weight gain in mice. Significant increases in white adipose tissue were observed in the HFD group compared to the Control group (*p* < 0.001), whereas GSE intervention significantly reduced this parameters compared to HFD-fed mice (*p* < 0.05). No statistically significant differences were observed in this index between the GPE and HFD groups, but exhibited a decreasing trend (Figure 2C). Histomorphological analysis revealed significant differences in tissue morphology between groups. H&E staining demonstrated that adipocytes in white adipose tissue (WAT) exhibited larger cross-sectional areas in high-fat diet-fed mice with no supplements compared to the other three groups (Figure 2B,D), and the hepatic lipid accumulation was significantly reduced after GSE and GPE treatment. Gross morphological examination revealed visibly darker brownish livers in the HFD group mice relative to other groups (Figure 2E). Figure 2F shows a significant increase in fat in the liver tissue of the HFD group compared to other groups. Both GSE and GPE supplementation significantly reduced the liver index (liver weight/body weight) relative to the HFD group, and the liver weight of mice in GSE group also decreased significantly (Figure 2G,H). Both GSE and GPE significantly reduced the hepatic TC levels (Figure 2J). GSE markedly decreased liver TG content and GPE treatment only showed a nonsignificant trend toward TG reduction (Figure 2I). In addition, both GSE and GPE effectively attenuated HFD-induced elevations in serum aspartate AST and ALT activities (Figure 2K,L).

### 3.2. Alpha Diversity

**Figure 3:**
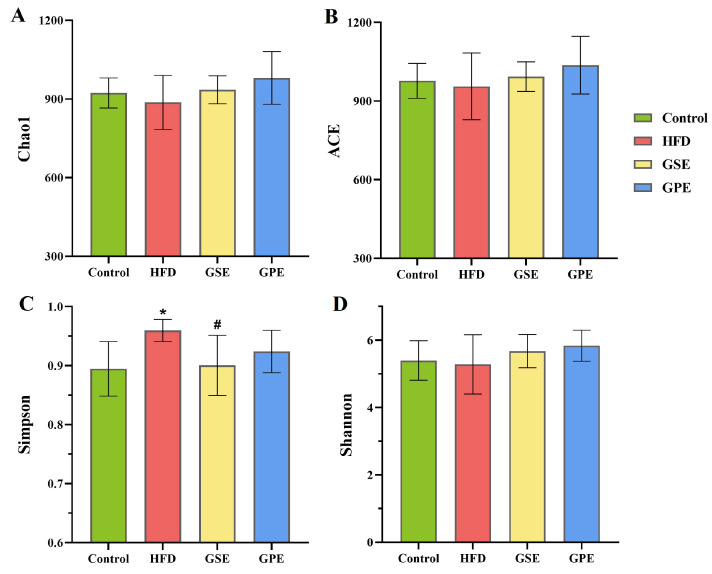
The effects of GSE and GPE on the α-diversity of gut microbiota. (**A**) Chao1 index. (**B**) ACE index. (**C**) Simpson index. (**D**) Shannon index. Data are presented as means ± SEMs, * *p* < 0.05 versus Control group, ^#^ *p* < 0.05 versus HFD group.

In alpha diversity, the Chao1 and ACE indices represent the abundance of microbes, while the Shannon and Simpson indices represent the abundance and evenness of the microbial community. Figure 3 demonstrates that only the Simpson index exhibited significantly decreased values in both Control and GSE groups relative to the HFD group (*p* < 0.05), whereas remaining alpha diversity indices showed no statistical differences between groups. However, all indices suggested a trend of lower diversity and abundance in the HFD group compared to the other groups. This indicates that the seed polyphenol extract from grape pomaces has a certain improvement on the evenness of intestinal microbiota abundance in mice, while the polyphenol extract from grape peels exhibits limited effectiveness in enhancing gut microbial diversity.

### 3.3. Beta Diversity

**Figure 4:**
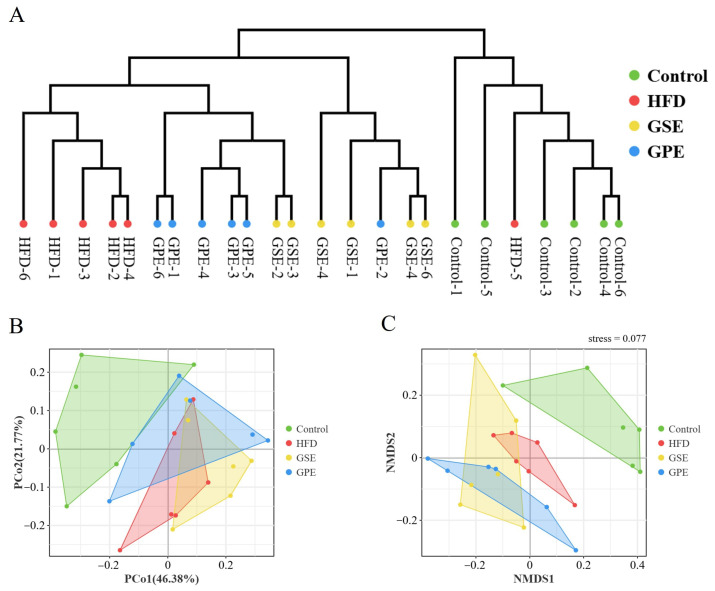
The effects of GSE and GPE on the β−diversity of gut microbiota. (**A**) The UPGMA cluster analysis. (**B**) PCoA analysis. (**C**) NMDS2 analysis.

The UPGMA cluster analysis of beta diversity index among samples was conducted at the OTU level, as shown in Figure 4A. The clustering tree demonstrates that the clustering results of samples are fundamentally consistent with experimental groupings. The experimental groups were stratified into two primary categories: those fed a standard diet and those receiving a high-fat diet. Despite eating a HFD diet, the GSE and GPE groups were significantly different from the HFD group.

The principal coordinates analysis (PCoA) is based on the distance matrix and the nonmetric multi-dimensional scaling (NMDS) analysis is based on the ranking of distance between samples were performed, as shown in Figure 4B,C, respectively. Analysis revealed that the Control group exhibited a distinct separation from other groups. PCoA revealed partial overlaps among the sample distributions of the HFD, GSE, and GPE groups. In the NMDS analysis, the GSE group obviously overlaps with the GPE group, and the HFD group partially overlaps with the GSE group. These results demonstrate that the Control group exhibited significant differences in gut microbial communities compared to other groups. Both GSE and GPE induced distinct alterations in the gut microbiota of HFD-fed mice, with some degree of difference. The intergroup differences warrant further analysis.

### 3.4. Composition and Structure of Gut Microbiota

**Figure 5:**
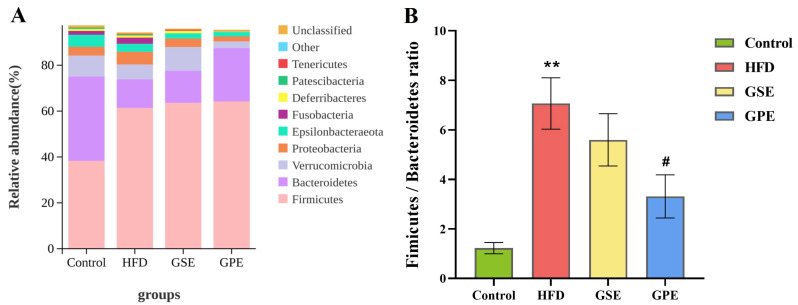
The effects of GSE and GPE on the structure of gut microbiota at the phylum level. (**A**) Stacking diagram at the phylum level. (**B**) Firmicutes/Bacteroidetes ratio. Data are presented as means ± SEMs, ** *p* < 0.01 versus Control group, ^#^ *p* < 0.05 versus HFD group.

At the phyla level, the gut microbiota in this study was primarily composed of the Firmicutes, Bacteroidetes, Verrucomicrobia, Proteobacteria, and Epsilonbacteraeota. Compared with other groups, the relative abundance of pathogenic bacteria Proteobacteria in HFD group was higher, as shown in Figure 5A.

Firmicutes and Bacteroidetes are the most important microorganisms in the gut. As a well-established indicator of microbial community homeostasis, the Firmicutes/Bacteroidetes (F/B) ratio provides critical insights into gut microbiota dysbiosis. The HFD group demonstrated a significantly higher F/B ratio compared to Control animals (*p* < 0.01), while GPE supplementation resulted in a significantly lower ratio relative to HFD (*p* < 0.05). There was no significant intergroup variation in F/B ratios between GSE and HFD groups, but the average F/B ratio of GPE was lower than that of the HFD group, as shown in Figure 5B. The above analyses indicate that GPE significantly ameliorates high-fat-diet-induced gut microbiota dysbiosis, and GSE also play this role to a certain extent.

### 3.5. Genus-Level Abundance of Mouse Gut Microbes

**Figure 6:**
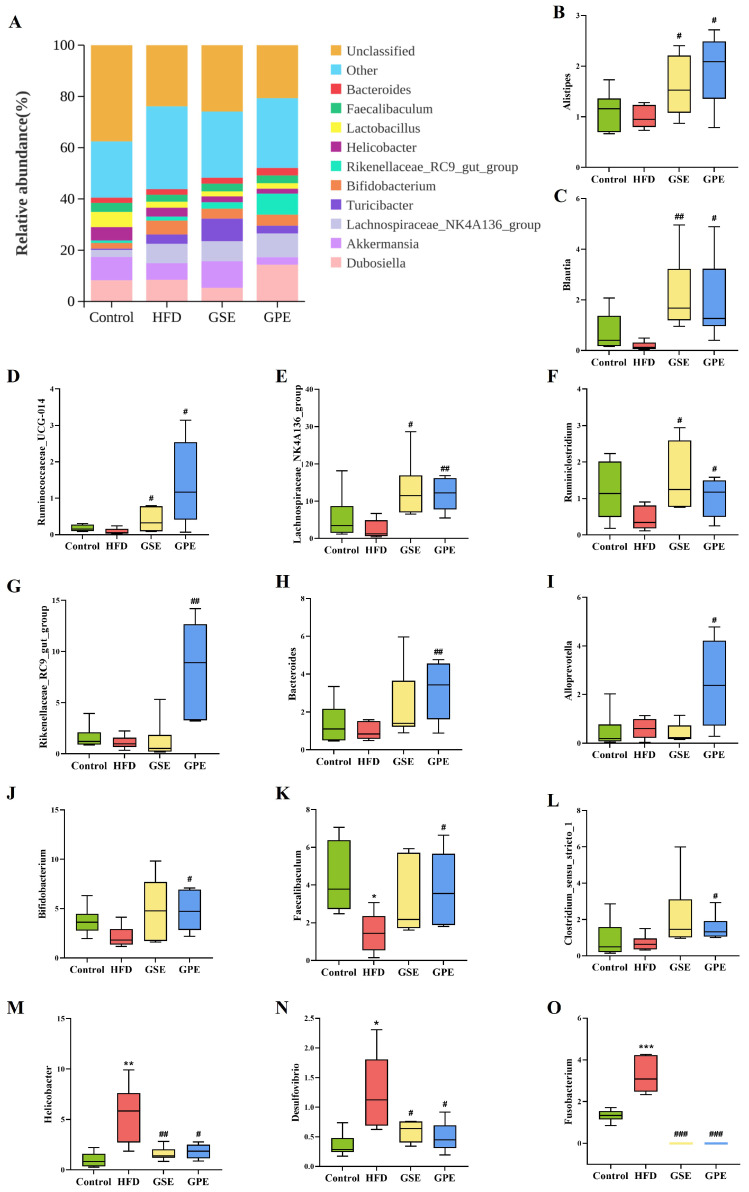
The effects of GSE and GPE on the gut microbiota at the genera level. (**A**) Stacking diagram at the genera level. Relative abundance changes of representative genera: (**B**) *Alistipes*, (**C**) *Blautia*, (**D**) *Ruminococcaceae_UCG_014*, (**E**) *Lachnospiraceae_NK4A136_group*, (**F**) *Ruminiclostridium*, (**G**) *Rikenellaceae-RC9-gut-group*, (**H**) *Bacteroides*, (**I**) *Alloprevotella*, (**J**) *Bifidobacterium*, (**K**) *Faecalibaculum*, (**L**) *Clostridium_sensu_stricto_1*, (**M**) *Helicobacter*, (**N**) *Desulfovibrio*, and (**O**) *Fusobacterium*. The box plot shows the median (center line), interquartile range (IQR, box bounds), and whiskers extending to the Min to Max, * *p* < 0.05, ** *p* < 0.01, *** *p* < 0.001 versus Control group, ^#^ *p* < 0.05, ^#^^#^ *p* < 0.01, ^#^^#^^#^ *p* < 0.001 versus HFD group.

At the genus level, the predominant gut microbiota in mice identified in this study included *Demoiselle*, *Akkermansia*, *Lachnospiraceae_NK4A136_group*, *Turicibacter*, *Bifidobacterium*, *Rikenellaceae_RC9_gut_group*, *Helicobacter*, *Lactobacillus*, and *Faecalibaculum* (Figure 6A).

To further investigate the differences details of gut microbiota among the groups, analysis of predominant bacteria was conducted at the genus level. Mice in the GSE and GPE groups exhibited significantly higher abundances of *Alistipes*, *Blautia*, *Ruminococcaceae_UCG014*, *Lachnospiraceae_NK4A136_group*, and *Ruminiclostridium* in comparison with the HFD group. Abundance levels of *Rikenellaceae-RC9-gut-group*, *Bacteroides*, *Alloprevotella*, *Bifidobacterium*, *Faecalibaculum*, and *Clostridium-sensu-stricto-1* in the GPE group were significantly higher than those in the HFD group. High-fat diet increased the abundance of *Helicobacter*, *Desulfovibrio*, and *Fusobacterium*, which could be prevented by feeding GSE and GPE (Figure 6B–O).

### 3.6. Biomarkers in Different Groups

**Figure 7:**
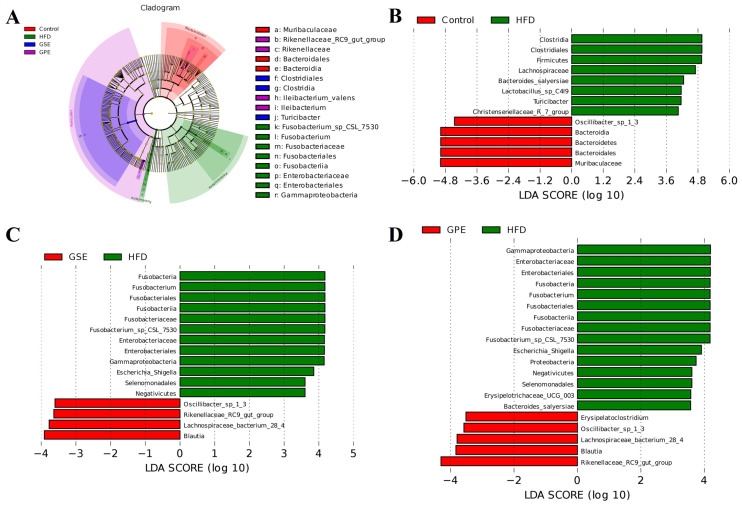
The effects of GSE and GPE on the gut microbial community in HFD-fed mice. (**A**) LDA Effect Size (LEfSe) analysis. (**B**) Linear Discriminant Analysis (LDA) for HFD vs. Control. (**C**) GSE vs. HFD. (**D**) GPE vs. HFD.

To further investigate the biomarkers of gut microbiota in each group of mice fed a HFD, GSE, and GPE in this study, differential gut microbiota screening across the four groups was conducted through Linear Discriminant Analysis (LDA) and LDA Effect Size (LEfSe). The LEfSe result (Figure 7A) revealed that the dominant microorganisms in the HFD group were enriched with Proteobacteria phylum, while the Control group’s dominant microorganisms were enriched with Bacteroidetes phylum. The predominant microorganisms in both the GSE and GPE groups exhibited substantial overlap, with a significant enrichment observed within the Firmicutes phylum.

We compared the gut microbiota of each group in pairs; the result revealed that HFD-fed mice showed greater Firmicutes abundance and reduced Bacteroidetes levels compared to the Control group (Figure 7B), which is consistent with the phylum-level F/B ratio results. Additionally, the HFD group showed higher abundances of *Clostridia*, *Lachnospiraceae*, *Bacteroides_salyersiae*, *Lactobacillus_sp_C419*, *Turicibacter*, and *Christensenellaceae_R-7_group*, while the abundances of *Muribaculaceae* and *Oscillibacter* exhibited marked decreases compared to the Control group. Pairwise comparisons of gut microbiota composition demonstrated that, relative to Controls, the HFD group showed significant enrichment of Firmicutes alongside a decrease in Bacteroidetes (Figure 7C,D). The HFD group exhibited a significantly higher abundance of *Enterobacterales*, *Enterobacteriaceae*, *Fusobacteria*, *Escherichia-Shigella*, *Proteobacteria*, *Erysipelotrichaceae*, and *Bacteroides_salyersiae* compared to both the GSE and GPE groups (Figure 7C,D). GSE elevated abundances of *Oscillibacter*, *Rikenellaceae_RC9_gut_group*, *Lachnospiraceae_bacterium*, and Blautia relative to the HFD group. The biomarker in the GPE group was basically consistent with those in the GSE group.

### 3.7. SCFA Content and Its Correlation Analysis with Gut Microbiota

**Figure 8:**
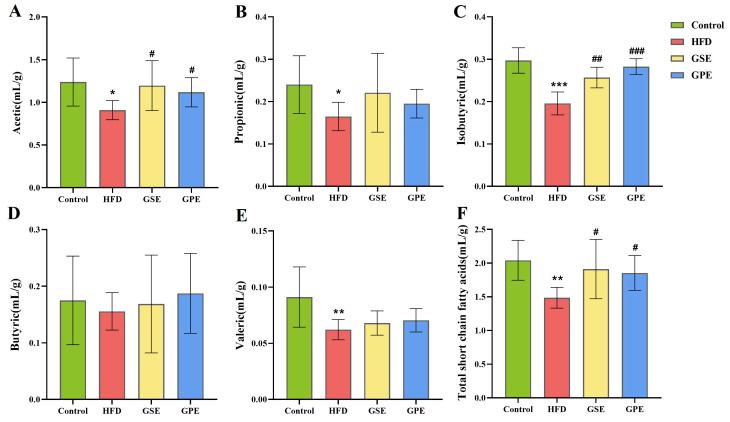
The effects of GSE and GPE on colonic SCFA. (**A**) Acetic. (**B**) Propionic. (**C**) Isobutyric. (**D**) Butyric. (**E**) Valeric. (**F**) Total SCFAs. Data are presented as means ± SEMs, * *p* < 0.05, ** *p* < 0.01, *** *p* < 0.001 versus Control group, ^#^ *p* < 0.05, ^#^^#^ *p* < 0.01, ^#^^#^^#^ *p* < 0.001 versus HFD group.

SCFAs are metabolic products of gut microbiota, which not only have a close relationship with gut microbiota but also influence glucose and lipid metabolism [19]. We measured the levels of various SCFAs and total SCFA content in the colon. Analysis revealed markedly decreased concentrations of all SCFAs in the HFD group versus Control group. In contrast, GSE and GPE interventions substantially elevated acetic acid, isobutyric acid, and total SCFA levels compared to HFD (Figure 8). The reduction in SCFAs caused by a high-fat diet could be prevented by feeding mice GSE and GPE.

**Figure 9:**
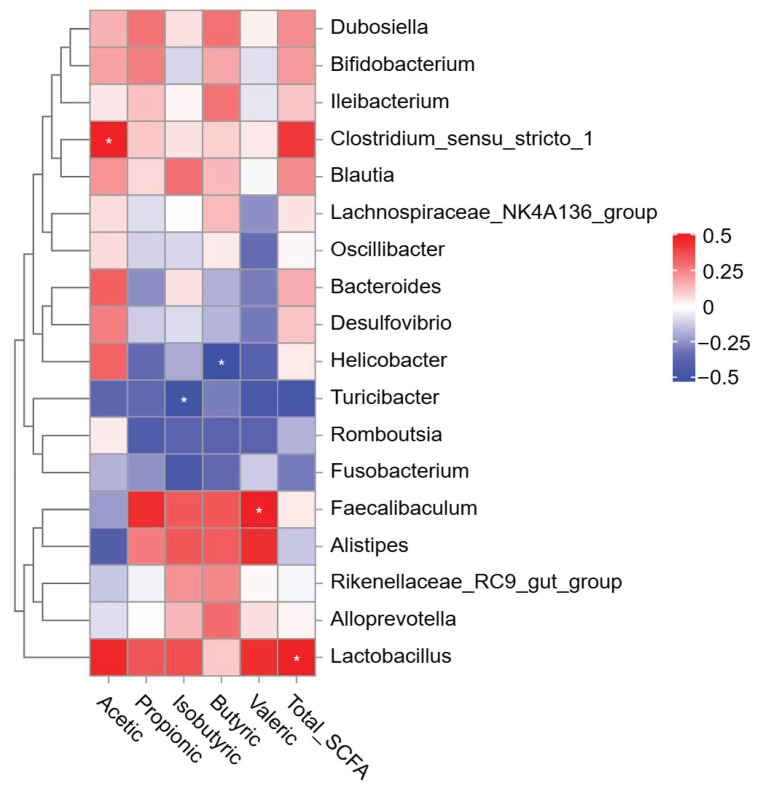
Pearson’s correlation coefficients between SCFAs and key gut microbes in Control, HFD, GSE, and GPE groups. |r| > 0.5 suggests significant correlation between gut microbes and SCFA represented by *.

SCFAs are metabolites produced by gut microbiota. The correlation analysis was conducted to understand the relationship between gut microbiota and SCFAs under GSE and GPE treatments. The results showed that (Figure 9) *Helicobacter* and *Turicibacter* were significantly negatively correlated with butyric and isobutyric acids. The correlation analysis revealed significant positive associations between: (i) *Lactobacillus* abundance and total SCFA concentrations, (ii) *Faecalibaculum* and valeric acid levels, and (iii) *Clostridium-sensu-stricto-1* and acetate. *Helicobacter* and *Turicibacter* were significantly negatively correlated with butyric and isobutyric acids. Additionally, *Lactobacillus*, *Ileibacterium*, *Alistipes*, *Dubosiella*, *Bifidobacterium*, and *Blautia* exhibited varying degrees of positive correlation trends with different SCFAs, suggesting that these bacteria may be directly or indirectly related to SCFA production. It is possible that they are SCFA producers.

## 4. Discussion

### 4.1. Grape Pomace Polyphenols Reduce Obesity

Polyphenols exhibit well-characterized pleiotropic bioactivities, including anti-inflammatory and antioxidant activities and modulation of glucose and lipid homeostasis [8]. These factors affect changes in weight, and body weight, adipose tissue weight, and liver weight can intuitively indicate the degree of obesity. We found that although the polyphenols contained in grape pomace have limited bioavailability [5], they still have biological activity in alleviating obesity.

Our results indicate that grape pomace polyphenols can effectively improve high-fat-diet-induced obesity in mice. GSE and GPE can effectively reduce the increase of body weight, white adipose tissue, hepatic lipid accumulation, and liver function index induced by a high-fat diet (Figure 2). A study on tea polyphenols demonstrated that Fu instant tea alleviated fatty liver disease and significantly reduced both body weight and liver weight in HFD-fed mice [20]. Although the main polyphenol components of grapes and tea are not exactly the same, our results indicate that grape pomace polyphenols can effectively improve obesity in mice on a high-fat diet. Furthermore, a study has shown that grape polyphenols can effectively ameliorate high-fat-diet-induced metabolic disorders [21], and the results of the present study are consistent with this result.

### 4.2. GSE and GPE Affect Gut Microbial Structure

Alpha diversity serves as the primary metric for evaluating gut microbiota profiles, quantifying both species abundance and community evenness. The present findings revealed that the HFD group exhibited a slight reduction in gut microbiota abundance and evenness, though the analysis did not reveal any significant statistical distinctions except the Simpson index (Figure 3). Specific functional components drive structural changes within the gut microbiota ecosystem, such as resveratrol and Ce-RS3, which can significantly increase the diversity of the gut microbiota in obese mice [22,23]. Our findings suggest that regulating microbiota alpha diversity is not a major way for GPE and GSE to improve obesity.

Both hierarchical clustering and dimensionality reduction analysis are ways to intuitively reflect the differences between groups. Although GSE and GPE administration did not significantly affect α-diversity indices, β-diversity demonstrated significant separation between the two groups treated with grape pomace polyphenol and the HFD group. The HFD diet was decisive for the alteration of gut microbiota, while GSE and GPE could regulate the gut microbiota, which had a great degree of similarity (Figure 4).

At the phylum level, Firmicutes and Bacteroidetes constitute the most predominant components of the gut microbiota. Because of their high relative abundance, changes in them can directly affect the gut microbiota as a whole. The ratio of Fimicutes to Bacteroidetes (F/B) is widely regarded as a critical indicator of the status of gut microbiota as well as a response to the health of the host. In the current study (Figure 5B), GPE treatment downregulated the F/B ratio in the HFD mice (*p* < 0.05). In [22], the F/B ratio was elevated in obese mice and decreased after treatment with Type 3 Resistant Starch derived from Canna edulis, which ameliorated mice obesity. The results of the present study are consistent with this finding. GPE demonstrated superior regulatory efficacy compared to GSE regarding this index.

### 4.3. Intake of GSE and GPE Altered Biomarkers in HFD Mice

LDA and LEfSe analysis results revealed significant differences in gut microbiotal species between the HFD group and the Control group. Enrichment of probiotics was used as biomarkers in the mice of the GSE and GPE groups. *Blautia*, *Rikenellaceae RC9_gu_group*, *Lachnospiraceae_bacterium_28_4*, *Oscillibacter_sp_1_3*, and *Erysipelatoclostridium* were enriched in the GSE and GPE groups (Figure 7). Based on genus-level relative abundance analysis, we identified specific microbial compositional differences among groups. These differences and their potential physiological implications were subsequently analyzed as follows.

*Blautia* has garnered significant attention from researchers due to its potential contributions to alleviating inflammatory and metabolic diseases [24]. The LDA results of this study align with the genus-level relative abundance analysis. It indicated the increasing abundance of *Blautia* in both GSE and GPE groups compared with the HFD group (Figure 6C and Figure 7C,D). It has been found to be significantly negatively correlated with visceral fat mass and the severity of alcoholic fatty liver disease [25,26]. It has been reported that resveratrol feeding can increase the abundance of *Blautia* and decrease the abundance of *Desulfovibrio* in HFD mice [23]. A negative correlation between Blautia abundance and indicator markers of obesity-related metabolic disorders has also been reported [27]. The findings in this study align with the results of reports mentioned above. *Lachnospiraceae bacterium 28-4* play an important role in reducing food intake, attenuating HFD-induced weight gain, and improving insulin resistance in mice [28]. In the present study, obesity was prevented in GSE and GPE mice, and there was a trend toward higher relative abundance of *Lachnospiraceae bacterium 28-4* in the colon (Figure 7C,D). The results presented here support previous studies. *Bifidobacterium* is one of the most widely studied probiotic bacteria. The abundance of *Bifidobacterium* has been reported to be lower in obese individuals than in normal-weight individuals [29]. In this study, GPE increased the abundance of *Bifidobacterium* (Figure 6J).

Both LDA and genus-level relative abundance analysis demonstrated that GPE and GSE supplementation exerted positive modulatory effects on key beneficial genera, including *Blautia*, *Lachnospiraceae bacterium 28-4*, and *Bifidobacterium*. Notably, these microbial alterations showed correlations with SCFA production, as will be discussed in subsequent sections.

From previous reports, it can be inferred that relative abundance of Rikenellaceae is lower in obese individuals [30]. The relative abundance results in this study indicated that GPE significantly increased the abundance of *Rikenellaceae_RC9_gut_group* in HFD mice, while GSE did not (Figure 6G). Notably, *Rikenellaceae_RC9_gut_group* was enriched in both GSE and GPE groups in the LDA results (Figure 7C,D). A study on whether Myricetin alleviates high-fat-diet-induced atherosclerosis showed that Myricetin increased the abundance of *Rikenellaceae_RC9_gut_group* and alleviated atherosclerosis in mice of the high-fat diet group [31]. This suggests that grape pomace polyphenols, like other plant polyphenols, can increase the abundance of *Rikenellaceae_RC9_gut_group* and alleviate obesity. Additionally, it is interesting that GPE exerts stronger modulatory effects on *Rikenellaceae_RC9_gut_group* populations compared to GSE. A lower relative abundance of the *Oscillibacter* genus has been reported in individuals with NAFLD compared to healthy individuals [32]. In this paper, *Oscillibacter_sp_1_3* was enriched in both GSE- and GPE-treated groups compared with the HFD group (Figure 7C,D), suggesting that *Oscillibacter_sp_1_3* may be negatively associated with high-fat-induced liver injury and obesity. This may be related to the fact that *Oscillibacter* has the ability to metabolize cholesterol [33], which deserves further attention. *Rikenellaceae_rc9_gut_group* and *Oscillibacter_sp_1_3* are probiotics that have positive regulatory effects in the process of alleviating obesity by GSE or GPE.

*Erysipelatoclostridium* has been reported to decrease in abundance with improvements in pulmonary fibrosis and bile acid metabolism [34], while *Erysipelatoclostridiaceae* exhibits elevated abundance in individuals with obesity and inflammation [35]. In the present study, the enrichment of *Erysipelatoclostridium* in the GPE group suggests that GPE possibly exerts some negative effects on mice, which may also be related to individual differences (Figure 7D). Whether there are potential problems in the mechanism by which GPE regulates obesity needs to be further explored.

*Helicobacter* is an endotoxin-producing Gram negative bacterium that can cause gastritis, and reduction of Helicobacter can prevent fatty liver disease [36]. It has also been found that apple pomace has an inhibitory effect on *Helicobacter* [37]. *Desulfovibrio* are pathogenic bacteria, and several genera of the Desulfovibrionaceae family are considered opportunistic pathogens that cause inflammatory diseases [38,39]. *Fusobacterium* utilizes glutamate and lysine pathways to release harmful byproducts [40]. This study further confirmed the positive association between these bacteria and obesity and also indicated that GSE and GPE could exert beneficial effects on gut microbiota in HFD-fed mice (Figure 6M–O).

Genus-level profiling showed GPE increased probiotic abundances more than GSE (Figure 6); however, in the β-diversity cluster diagram, GSE showed greater β-diversity separation from HFD than GPE. In terms of physiological indicators, GSE can also significantly regulate the effects of HFD diet compared with GPE. While both extracts ameliorated diet-induced obesity and microbial dysbiosis, GSE exhibited superior overall efficacy, whereas GPE showed more pronounced effects on probiotic enrichment. These differential therapeutic profiles warrant further investigation.

### 4.4. Intake of GSE and GPE Elevated Colonic SCFA and the Relationship Between SCFA and Gut Microbiota

SCFAs are a crucial class of metabolites in the gut, produced by certain populations of gut microbiota through metabolizing dietary fiber [41]. They are not only absorbed through the intestinal epithelium metabolized as nutrients to supply energy for the body, but also play a part as signaling molecules to improve anemia, brain development, colorectal cancer, depression, obesity, and diabetes, as has been shown in more studies in recent years [42]. SCFA may be one of the pivots in the health effects of polyphenols, and these pathways tend to respond more rapidly in metabolic, neurological terms. We observed that mice fed with GSE and GPE exhibited higher levels of various SCFAs in the colon compared to the HFD group, with total SCFA levels significantly upregulated (Figure 8). This consists with the research results of Francyeli A. Silva’s in vitro fermentation with grape by-products [17]. In this study, GSE and GPE showed the most significant regulation of acetic acid and isobutyric acid in the colon of HFD mice; however, other SCFAs of GSE and GPE were not significantly different from HFD, but also showed a trend of elevated content. In a study on the effects of grape polyphenol and fiber-rich foods on the intestinal environment, it was found that different SCFA levels are regulated to different extents [17]. This may be attributed to the influence of distinct feeding materials on specific microbial species, leading to differences in their metabolic outputs.

It has been reported that *Blautia* produces acetic acid, ethanol, succinic acid, and lactic acid through fermentation [24]. The *Bifidobacterium* genus, which accounts for a relatively high proportion in the gut, is the most abundant genus within the Actinobacteria phylum, and it has been shown to produce acetate [43]. Acetate promotes the growth of bacteria that produce propionate and butyrate, while butyrate supports the proliferation of *Bifidobacterium*, which creates cross-feeding between bacteria. Members of the family Lachnospiraceae have been reported as major butyrate producers. This study revealed a positive correlation trend between the *Lachnospiraceae_NK4A136_group* and butyric acid levels, consistent with previous research [44]. Integrated analysis of correlation heatmap, genus-level relative abundance, and LDA results suggest that dietary supplementation with GSE and GPE significantly enriched the relative abundance of *Blautia*, *Bifidobacterium*, and *Lachnospiraceae_NK4A136_group* (*p* < 0.05). This microbial modulation may enhance SCFA production, contributing to alleviated obesity in mice.

*Faecalibacterium* strains primarily produce formate, small amounts of D-lactate, and significant quantities of butyrate during glucose fermentation [45]. In this study, *Faecalibacterium* showed a positive correlation trend with butyric acid and a significant positive correlation with valeric acid (*p* < 0.05), suggesting that butyric acid may promote valeric acid production. Existing evidence indicates that samples from lean individuals are enriched with more *Faecalibacterium* compared to those from obese and type 2 diabetes patients [46]. The results of this paper support this conclusion.

*Clostridium_sensu_stricto_1* are a group of mostly anaerobic bacteria [47], with acetic acid being a key metabolite, alongside other metabolites, such as butyric acid and lactic acid [48]. In a study by Xiaoqin Li et al. [49], the abundance of *Clostridium_sensu_stricto_1* increased after synbiotic supplementation, accompanied by enriched pathways for acetic acid metabolism. Association analysis in this study revealed a highly positive correlation between acetic acid levels and *Clostridium_sensu_stricto_1* (Figure 9), which is consistent with Li’s study. This provides evidence that *Clostridium_sensu_stricto_1* regulates host health through acetic acid production.

According to current research reports, the species *Alistipes putredinis* can generate acetic acid, propionic acid, isobutyric acid, and isovaleric acid [50]. However, our findings revealed that *Alistipes* exhibited positive correlations with propionate, butyrate, isobutyrate, and valerate, while showing a negative correlation trend with acetate. This may be due to the different species of *Alistipes* in the two studies, resulting in differences in their correlation with SCFA. Reports on biomarkers of obesity and metabolic disorders indicate that *Alistipes* shows significant negative correlations with body weight, blood glucose, blood pressure, and blood lipid levels [51]. *Alistipes* have made certain contributions in reducing obesity, and it is likely that they alleviate obesity symptoms through SCFAs. Further research evidence is needed to determine which SCFAs *Alistipes* produce to improve obesity.

*Lactobacillus* has been reported to produce acetic, propionic, and butyric acids [52,53]. Association analysis in this study indicated that *Lactobacillus* had a positive trend of correlation with acetic, propionic, and butyric (Figure 9). Experimental evidence confirms that *Lactobacillus* alleviated HFD-induced mice obesity, potentially through affecting part of the fat metabolism [54]. In the above study, *Lactobacillus* as probiotics could not completely change the metabolic disorders, but had a modifying effect on them. Therefore, *Lactobacillus* may be regulating fat metabolism through the production of SCFA to reduce obesity, and the present study provides evidence to support this hypothesis.

This indicates that these SCFA producing bacteria play critical regulatory roles in alleviating obesity and relate metabolic disorders, and the mechanism of these action is to influence the metabolic process may through the production of SCFA. This study also provides new evidence for this speculation.

The present showed that GSE and GPE alleviated the obesity, optimized the composition of gut microbiota, and increased the fecal SCFA content in HFD mice. Combined with the existing studies, it can be speculated that SCFA may be one of the important factors to reduce obesity. Some researchers have also explored the mechanism of SCFA regulating obesity and metabolic disorders. In chronic inflammation, SCFA binds to receptors GPR41, GPR43, and GPR109A, inhibits cAMP-dependent signaling pathways, suppresses the NF-kB signaling pathway, and activates the mTOR signaling pathway, which attenuates the inflammatory response [55,56]. In terms of glucose metabolism, SCFA can protect the structure and function of pancreatic β cell through glucose like peptide-1 (GLP-1) [57], or promote the secretion of PYY to improve glucose metabolism [58]. In terms of lipid metabolism, SCFA accelerates the oxidation of lipids in liver and adipose tissue and reduces their synthesis, thereby reducing lipid accumulation [59].

GSE and GPE, abundantly available as by-products of wine production, have been demonstrated in this study to modulate gut microbiota composition and enhance short chain fatty acid (SCFA) production. Further investigation into the mechanistic basis of GSE and GPE antiobesity effects is critical for the development of novel nutraceutical or functional food interventions targeting metabolic disorders. Additionally, such research may contribute to the sustainable utilization of winemaking by-products.

## 5. Conclusions

This research assessed the impact of GSE and GPE on the gut microbial community and SCFAs in diet-induced obese mice, confirming their antiobesity potential against HFD effects. Interestingly, GSE and GPE regulate the microbial community by influence on the biomarker abundance instead of alpha diversity. The results suggested that GSE and GPE reduced the elevated F/B ratio caused by HFD to different degrees. GSE and GPE reduce the HFD-induced elevated relative abundance of pathogenic bacteria, especially Fusobacteria and Eschericha-Shigella. Meanwhile, they elevated the abundance of probiotics such as *Lachnospiraceae_NK4A136_group*, *Bifidobacterium*, and *Blautia*. Furthermore, *Lactobacillus*, *Faecalibaculum*, *Clostridium-sensu-stricto-1*, *Bifidobacterium*, *Blautia*, *Alistipes*, and *Dubosiella* may be regulated by GSE and GPE to produce SCFA, alleviating obesity. Our research results provide evidence to support the beneficial effect of grape pomace polyphenol extract in improving obesity of HFD mice.

## Data Availability

The original contributions presented in the study are included in the article. Further inquiries can be directed to the corresponding author.

## Appendix A

**Table A1:** AIN 93M ingredients.

Ingredients	Content (g/kg)
Soybean Oil	40
Sucrose	100
Maltodextrin	155
Casein	140
Cellulose	50
Vitamin Mix, V1010	10
Mineral Mix, M1021	35
Corn Starch	465.7
L Cystine	1.8
Choline Bitartrate	2.5
TBHQ	0.036
Total	1000

**Table A2:** TP23100 ingredients.

Ingredients	Content (g/kg)
Lard	196
Soybean Oil	30
Sucrose	202
Maltodextrin	125
Casein	175
Cellulose	62
Vitamin Mix, V1010	12
Mineral Mix, M1021	61
Corn Starch	132
L Cystine	2
Choline Bitartrate	3
TBHQ	0.045
Total	1000

**Table A3:** AIN 93M calories percentage.

	Calories Percentage (%)
Protein	14.1%
Carbohydrate	75.9%
Fat	10.0%

**Table A4:** TP23100 calories percentage.

	Calories Percentage (%)
Protein	14.1%
Carbohydrate	40.9%
Fat	45.0%

**Table A5:** Total phenolic (TP), total tannin (TAN), total flavonoid (TFO), total flavanol (TFA), and total anthocyanin (TA) content in GSE and GPE [5].

	TP (mg/g)	TAN (mg/g)	TFO (mg/g)	TFA (mg/g)	TA (mg/g)
GSE	656.40 ± 8.07	680.62 ± 3.82	271.20 ± 0.85	147.64 ± 1.78	-
GPE	173.70 ± 6.22	109.89 ± 1.66	105.53 ± 0.55	14.76 ± 0.54	75.46 ± 1.88
